# Nurse staffing models in acute care: A descriptive study

**DOI:** 10.1002/nop2.321

**Published:** 2019-06-17

**Authors:** Marianne Thériault, Carl‐Ardy Dubois, Roxane Borgès da Silva, Alexandre Prud’homme

**Affiliations:** ^1^ Faculty of Nursing University of Montreal Montreal Quebec Canada; ^2^ Public Health Research Institute University of Montreal Montreal Quebec Canada; ^3^ Department of Management, Evaluation and Health Policy University of Montreal Montreal Quebec Canada

**Keywords:** acute care, administration, groups, nurses, nursing, skill mix, staff mix, staffing

## Abstract

**Aims:**

To identify nurse staffing groups in acute care facilities.

**Design:**

This retrospective descriptive study used a configurational approach.

**Methods:**

Data from a two‐month target period from January–March 2016 were collected for 40 facilities in four different hospitals in one of the largest regions of Quebec. Multiple factorial analysis and hierarchical ascendant classification were used to generate a limited number of nurse staffing groups.

**Results/Findings:**

Four distinct nurse staffing groups emerged from this study. The least resourced model relied mainly on less qualified personnel and agency staff. The moderately resourced basic model was assessed as average across all staffing dimensions, but employed less overtime, relying mostly on auxiliary nurses. The moderately resourced professional group, also moderate in most variables, involved more overtime and fewer less qualified personnel. The most resourced group maximized highly qualified personnel and minimized instability in the nursing team.

**Conclusion:**

This study covered multiple staffing groups with widely varying characteristics. Most groups entailed risks for quality of care at one or more levels. Few care units approached the theoretical staffing ideal.

## 1 INTRODUCTION

Staffing is a cornerstone of human resource management. The performance of any healthcare organization depends primarily on the continuous availability of enough qualified workers, judiciously deployed and operating in a work environment that enhances their productivity. Studies showed the importance of adequate staffing for optimizing both patient outcomes and the quality and security of care.

In many industrialized countries, healthcare systems are facing a rising demand for hospital care (Australian Institute of Health & Welfare, 2013; Kork & Vakkuri, 2016; Ordre des infirmières et infirmiers du Québec [OIIQ], 2014a; OIIQ, 2014b; Papi, Pontecorvi, & Setola, 2016), while simultaneously dealing with a decrease in the number of nurses able to provide care (Kork & Vakkuri, 2016; Needleman, 2015; OIIQ, 2014a).

Many hospitals in industrialized countries are understaffed due to instability among staff, caused partly by high turnover rates (Hayes et al., 2012; O'Brien‐Pallas, Murphy, Shamian, Li, & Hayes, 2010) and high absenteeism rates (Murphy et al., 2012). In 2010, the overall nurse turnover rate for Canada was 19.9% (O'Brien‐Pallas et al., 2010), which leads to increased workload for the remaining nurses (Duffield et al., 2011; Twigg, Duffield, Bremner, Rapley, & Finn, 2011).

Similarly to other jurisdictions, the staffing problem can also be observed in the province of Quebec, Canada, where hospitals have a 100% occupancy rate, causing longer emergency room stays (OIIQ, 2014b). As hospitals must provide continuous care for more patients while maintaining high levels of quality and safety of care, they face significant challenges with respect to nurse staffing.

The repercussions are felt by everyone involved as follows: patients, nurses and healthcare organizations. Nurse staffing problems have been linked to higher patient mortality rates (Aiken, Sloane, Bruyneel, Achterberg, & Sermeus, 2014; Needleman et al., 2011) and an increase in inpatient adverse events such as falls and medication errors (Ministère de la santé et des services sociaux du Québec [MSSS], 2015). The consequences for nurses include job dissatisfaction, burnout (Duvall & Andrews, 2010), more frequent work‐related accidents (Bae, 2013), greater workload (Needleman, 2015; OIIQ, 2014b) and higher overtime rates (Bae, 2013; OIIQ, 2014c). Nurse staffing problems also have an impact on health organizations by significantly increasing related costs (Hill, Higdon, Porter, Rutland, & Vela, 2015; Martsolf et al., 2014).

There are no clear and established practices to ensure optimal, efficient and realistic nurse staffing (Needleman, 2015). Moreover, there are many different interpretations of nurse staffing (Spetz, Donaldson, Aydin, & Brown, 2008), depending on the deployment, quantity and qualifications of the available workforce and on work conditions.

Given the multidimensional aspect of nurse staffing, most previous studies have taken widely varying directions or used a narrow approach, focusing on one dimension of staffing above others. This typically results in a fragmented vision that does not reflect the full extent and complexity of the issue. One possible explanation for this is the wide range of variables affecting staffing (Spetz et al., 2008), which give rise to diverse definitions of the concept itself.

Despite the abundance of literature on nurse staffing, there is persisting knowledge gap concerning empirical configurations and forms of staffing in different contexts of care. Hospitals do not have standardized methods for collecting data, which can be gathered variably by individual units, groups of care units or by hospitals (Spetz et al., 2008), according to different factors such as type, number or composition of nursing teams (Dubois et al., 2012; Schell, 2014). The data are generally systematically collected by payroll departments (Spetz et al., 2008), but is not systematically processed and analysed from a staffing perspective. Therefore, managers do not get clear feedback about the staffing groups that result from their recruitment and human resource deployment decisions.

This study proposes to address knowledge gaps concerning nursing staffing groups through a thorough analysis of care units’ staffing characteristics from a multidimensional perspective in the context of one of the largest regions in Quebec.

## 2 BACKGROUND

Numerous recent empirical studies have associated nurse staffing with patient outcomes (Aiken et al., 2014; Bae, 2013; Dall, Chen, Seifert, Maddox, & Hogan, 2009; Lobo, Fisher, Ploeg, Peachey, & Akhtar‐Danesh, 2013; Needleman et al., 2011). In these studies, nurse staffing is discussed in terms of three staffing dimensions: resource levels, staff mix and stability of the team.

### 2.1 Resource levels

This dimension refers to the availability of a sufficient workforce to provide services to all levels of the organization (care unit, type of unit, hospital). It also reflects the efficiency of original planning, staffing deployment, attraction and retention of personnel, turnover rates, etc.

This dimension is operationalized by daily patient‐to‐nursing staff ratios, hours of direct patient care by nursing staff, nurse workloads, overtime use and full‐time equivalents, among others. The first two measures are typically obtained by dividing a volume of patients or a quantity of patient care services by a volume of nurses, and they reflect the ability to align nursing resources with service demands. Nurse workload is a very complex concept, which is linked to patient–nurse ratios (Duffield et al., 2011). As overtime represents a palliative measure to manage lack of personnel, measuring the number of voluntary and mandatory overtime hours is also an indirect method of assessing the workforce deployed on a care unit (Bae, 2013; Lobo et al., 2013).

Considered independently, this dimension faces multiple limitations. Measures of available workforce often expressed in full‐time equivalents or number of staff members (Dubois & Singh, 2009; Duffield et al., 2011) do not give any detail on workforce characteristics. The patient–nurse ratio links the workforce and patients in the facility but, on its own, does not offer information on levels of patient acuity (Murphy et al., 2012). Even more critically, it can be difficult to obtain data in the first place, since existing tools to examine workload, such as the PRN 80, Nursing Activities Score (NAS) or OMEGA (Carayon & Gürses, 2005), are complex systems providing exhaustive assessments (Tilquin, 2003). Finally, it is just about impossible to design a classification system or a valid score for all contexts of care and all clienteles (Tilquin, 2003).

### 2.2 Staff mix

The staff mix dimension goes beyond staffing numbers to focus on the composition of the care team, especially the nursing team. It reflects the extent to which team members possess the abilities and expertise required to meet care needs.

The operationalization of this dimension generally relies on four variables: the types of nurses on staff (Butler et al., 2011), their number of years of experience (Butler et al., 2011; Gerdtz & Nelson, 2007; McGillis Hall, Doran, & Pink, 2004), team interdisciplinarity and the presence of support staff. Determining the types of nurses on the team involves differentiating personnel according to their education. Interdisciplinarity refers to the degree to which several different types of professionals are mobilized within a care team. The presence of support staff can increase the efficiency of the nursing team, by freeing team members to focus on nursing tasks rather than administrative duties.

The staff mix dimension also has limitations. Evaluating the types of nurses on a team based on their education emphasizes school degrees rather than their expertise. The presence of support staff was noted as a variable in the literature, but no precise measure was identified. Nurses’ years of experience do not always represent the extent of their professional careers or significant events. Finally, interdisciplinarity is often hard to define due to the complexity of assessing it.

### 2.3 Team stability

This dimension refers to the ability to maximize labour participation out of the available workforce and to the conditions that ensure staff retention. Staff retention is often linked to an optimal work environment for nurses (Aiken, Clarke, Sloane, Lake, & Cheney, 2008) and to work satisfaction. Work satisfaction is partly related to the level of stress in the work environment, patient satisfaction and the quality of care delivered (Gagné, Laforest, & Lavoie‐Tremblay, 2003).

This dimension is generally operationalized by five variables: workforce status, absenteeism (and the staffing replacement it necessitates), use of float teams, supplemental use of agency staff and nurse turnover. Workforce status, represented by the permanency of positions and often measured in terms of percentages of positions of different status (OIIQ, 2014d), indicates the ability to use the available workforce pool and the level of job insecurity. Absenteeism is often measured by absenteeism rates or hours of absenteeism (Canadian Federation of Nurses Unions [CFNU], 2015; Murphy et al., 2012). It is associated with lack of supervisor support, increased workload (Murphy et al., 2012; Rajbhandary & Basu, 2010) and a stressful work environment (Peate, 2015; Rajbhandaray & Basu, 2010). The use of agency nurses, float nursing teams or other staff replacement mechanisms are other indicators of staffing instability. These variables can be measured in terms of percentages of hours worked by agency or float nurses (MSSS, 2014), among others. The turnover rate provides information on the difficulty of maintaining staff in positions.

The primary limitation shared by the variables for this dimension is that, taken separately, they do not provide detailed information on nurse staffing and are often the result of problems in other dimensions of staffing. Moreover, according to Duffield et al. (2011), workforce status is only raw data point that is not of the most relevant for understanding of team stability.

### 2.4 Framework

**Figure 1:**
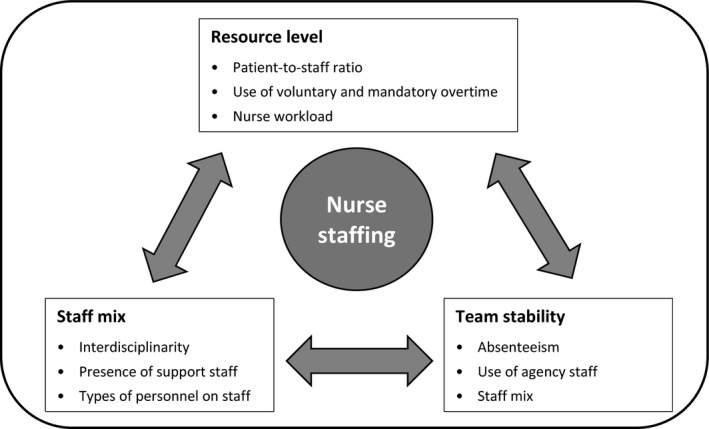
Nurse staffing conceptual model adapted from the American Nursing Association (1999), the Canadian Nurse Association (2005), Dubois and Singh (2009), and Duffield et al. (2011)

In sum, staffing can be conceptualized in three different ways, each one represented by a dimension of nurse staffing. These dimensions, presented in our conceptual model (Figure 1), are mutually complementary, as they each cover different aspects of the staff providing patient care. To the authors’ knowledge, there has not been any scientific research to date that combines all of these staffing dimensions.

In this conceptual framework, the three staffing dimensions are linked according to an open hypothesis that assumes that everything is connected and that each dimension interacts with the others, as shown in Figure 1. The variables used to operationalize each dimension in our study were established based on our interpretation of theoretical and empirical knowledge and on the limitations of each variable as described in the Background Section.

## 3 THE STUDY

### 3.1 Aim

The aim of the study was to identify nurse staffing groups in acute care facilities in one of the largest regions of the province of Quebec, Canada. The objective was to evaluate staffing in a multidimensional manner while simultaneously considering multiple variables pertaining to nurse staffing. This study enabled the authors to answer two research questions: What were nurse staffing configurations in acute care facilities in a region? And what were the characteristics of those configurations?

### 3.2 Design

The study design was descriptive exploratory research based on a configurational approach, which is commonly used to analyse multidimensional work. The configurational approach is defined by Meyer, Tsui, and Hinings (1993) as a “multidimensional constellation of conceptually distinct characteristics that commonly occur together.” This analytical approach generates groups composed of organizations that have similar conceptual characteristics (Cadez & Guilding, 2012; Fiss, 2007; Meyer et al., 1993). It also makes it possible to consider all three dimensions of nurse staffing concurrently, offering a more complete view of the concept. The data were collected retrospectively over a two‐month target period.

### 3.3 Participants

The target population of this study consisted of all the acute care facilities of a region. The region was selected through purposive sampling based on its five associated hospital centres and high number of acute care units. For this study, acute care facilities were defined as short‐term hospitalization units. The inclusion criteria were as follows:

- Being a short‐term hospitalization care unit belonging to a primary or secondary hospital centre in the chosen region;
- Belonging to a hospital centre that used the region's database to enter all staffing data and patient censuses electronically.

The authors chose to study the acute care facilities of a region as it offered a high diversity of care contexts in a single organization. One of the hospital centres in the studied region was not yet using the hospitals’ databases for patient censuses and was, therefore, excluded from the study. All tertiary care hospitals, long‐term hospitalization units, rehabilitation facilities and facilities where patients were not hospitalized were excluded, since they would have unnecessarily increased the sample's heterogeneity due to their significantly different staffing features. Facilities not offering acute care were likewise excluded due to their different characteristics that might not be comparable to others.

Since this study did not rely on parametrical statistics, it was not necessary to compute a precise sample size. However, Durand (2003) specifies that there should be a minimum of five individuals per variable, which in this study meant at least 40 care units in total to investigate eight variables. Ultimately, including all care units that met the inclusion criteria, the sample consisted of 40 acute care units in all, from four different hospital centres in the studied region.

### 3.4 Data collection

Data were collected by the first author from June to September 2016 directly from the human resource department (HRD) of the studied region. The target period for data collection was 11 January to 7 March 2016. These two months of data collection were chosen during a period of normal operation of the hospital to avoid the effects of seasonality (i.e. holidays).

The data collection grid was constructed by the authors according to the conceptual framework. The grid was based on the variables of the three dimensions of nurse staffing, broken down by their different indicators. Its aim was to capture administrative data relevant to the description of staffing characteristics in the care units. The grid included 26 items to collect for all three shifts (day, evening and night) for every day of the target period. The grid was subjected to a pre‐test conducted by the first author and a member of the HRD. The pre‐test allowed them to determine that the grid was correctly organized and would ensure efficient data collection. Unfortunately, data concerning interdisciplinarity and nurse workloads were not available during the collection period.

### 3.5 Validity, reliability and rigour

The data collection grid allowed to capture the administrative data required for this study. Thus, no psychometric evaluation of validity or reliability was required. The exploratory analyses relied on statistical methods, but also on interpretation of data (De Vaus, 2002) about nurse staffing. The selection of factorial axes and the choice of classification were based on the researchers’ interpretation of data in the light of theoretical and empirical knowledge of nurse staffing.

### 3.6 Construction of variables

All the variables studied in this research emerged from the three dimensions of staffing: resource levels, staff mix and team stability. Each variable under study was represented by its own indicator(s).

The resource level dimension was operationalized by two variables: patient–staff ratio and overtime use. The patient–staff ratio had two separate component indicators: the patient‐to‐nurse ratio and the ratio of patients to auxiliary nurses and nurse aides. Overtime use included three indicators: the proportion of overtime hours worked among three types of nurses: nurses without bachelor's degree, nurses with bachelor's degree and assistant head nurse. The staff mix dimension was operationalized by the proportions of these different types of nurses and the presence of support staff.

Team stability was operationalized by two variables: absenteeism and use of agency staff. Absenteeism encompassed three indicators corresponding to the three different types of nurses.

Details concerning how every indicator was constructed are listed along with their definitions in Table 1.

**Table 1:** Variable definitions and categories, by staffing dimension

Variables	Definition	Categories
Resource levels		
Patient‐to‐nurse ratio	Average number of patients during a day over the total working hours of RNs with and without bachelor's degrees during the same day (in patient/nursing hours)	Low (0–0.75) Average (0.76–1.5) High (more than 1.5)
Ratio of patients to auxiliary nurses and nurse aides	Average number of patients during a day over the total number of the nursing team during the same day (in patient/total number of the nursing team)	Low (0–4.9) Average (5.0–9.9) High (more than 9.9)
Overtime of RNs with bachelor's degree	Proportion of overtime hours worked by RNs with bachelor's degrees (in % of overtime hours/total of worked hours)	Low (0–4.9) High (5.0–12.0)
Overtime of RNs without bachelor's degree	Proportion of overtime hours worked by RNs without bachelor's degree (in % of overtime hours/total of worked hours)	Low (0–4.9) High (5.0–12.0)
Assistant head nurse overtime	Proportion of overtime hours worked by assistant head nurses (in % of overtime hours/total of worked hours)	Low (0–4.9) High (5.0–12.0)
Staff mix		
Proportion of RNs with bachelor's degree	Proportion of RNs with bachelor's degrees in the team (in % of total hours worked by RNs with bachelor's degrees/total hours worked by the nursing team)	Low (0–0.09) Average (0.10–0.249) High (0.25–0.40)
Proportion of RNs without bachelor's degree	Proportion of RNs without bachelor's degree in the team (in % of total hours worked by RNs without bachelor's degrees/total hours worked by the nursing team)	Low (0–0.449) Average (0.45–0.59) High (0.60–0.99)
Proportion of assistant head nurses	Proportion of assistant head nurses in the team (in % of total hours worked by assistant head nurses/total hours worked by the nursing team)	Low (0–0.09) Average (0.10–0.29) High (0.30–0.99)
Proportion of auxiliary nurses	Proportion of auxiliary nurses in the team (in % of number of auxiliary nurses/total number of the nursing team)	Low (0–0.19) Average (0.20–0.289) High (0.290–0.999)
Proportion of nurse aides	Proportion of nurse aides on the team (in % of number of nurse aides/total number of the nursing team)	Low (0–0.9) Average (0.10–0.29) High (0.30–0.99)
Support staff	Presence of support staff on weekdays during day shift	No Yes
Team stability		
Absenteeism of RNs with bachelor's degrees	Absenteeism ratio for RNs with bachelor's degrees (hours of absenteeism of RNs with bachelor's degrees/total hours worked by RNs with bachelor's degrees)	Low (0–4.9) Average (5.0–9.9) High (More than 9.9)
Absenteeism of RNs without bachelor's degree	Absenteeism ratio for RNs without bachelor's degree (hours of absenteeism of RNs without bachelor's degree/total hours worked hours by RNs without bachelor's degree)	Low (0–9.9) Average (10.0–19.9) High (More than 19.9)
Absenteeism of assistant head nurses	Absenteeism ratio for assistant head nurses (hours of assistant head nurse absenteeism/total hours worked by assistant head nurses)	Low (0–2.359) Average (2.360–4.99) High (More than 4.99)
Supplemental agency nurses	The care unit used supplemental agency nurses at least once during the data collecting period	No Yes

Abbreviation: RN, registered nurse.

The data were converted from continuous data to categorical variables to ensure they were comparable across units of care. The categorizations, also shown in Table 1, were established based on data distributions.

### 3.7 Ethical considerations

The authors obtained Research Ethics Committee approval from the University of Montreal's health research ethics board and the research ethics board of the healthcare centres in the region under study. The confidentiality of personal information was preserved by omitting names of both staff and patients. Moreover, only the authors had access to the data, which were locked in the office of the first author. The data will be conserved in a locked cabinet until seven years after the study, after which it will be destroyed.

## 4 DATA ANALYSIS

Our goal was to generate a classification of care units based on nurse staffing. Exploratory multidimensional statistics were carried out in two stages. First, we used multiple factorial analysis (MFA) (Bailey, 1994; Escofier & Pagès, 2008), a factorial analysis particularly appropriate for analysing variables grouped by dimension. MFA highlights the most significant structures of data (factors or factorial axes), in agreement with the initial concepts (Escofier & Pagès, 2008). Accordingly, the first MFA group was “resource levels,” the second group was “staff mix” and the third group was “team stability.” Three factorial axes were selected in the MFA, based on how well they each represented one of the groups (Kaufman & Rousseeuw, 2005), as evidenced by the groups’ respective contributions to these factorial axes (the first group contributed 61.4% to the third factorial axis, the second group 43.1% to the first factorial axis and the third group 54.4% to the second factorial axis).

**Figure 2:**
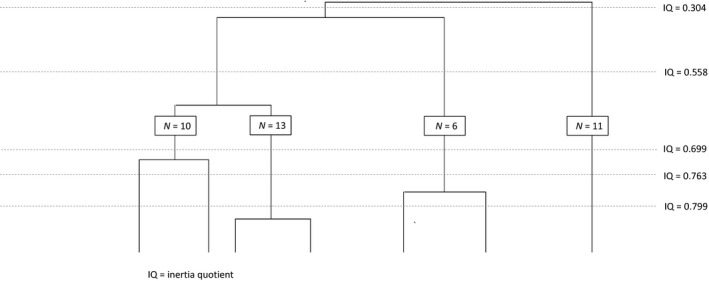
Dendrogram

In the second stage, we used ascending hierarchical classification (AHC) (Greenacre & Blasius, 1994) based on the three factorial axes retained. AHC is effective for partitioning groups by minimizing the internal variance of each class while maximizing variance between classes (Lebart, Morineau, & Piron, 2000). After examining the dendrogram and inertia quotients presented in Figure 2, we selected a four‐cluster partitioning. The dendrogram represented the arborescence of potential care unit clusters and the associated inertia quotients (inter inertia/total inertia) which are tending asymptotically to 1 (Borgès Da Silva et al., 2013). The homogeneity of the clusters increases as the inertia quotient increases. Hence, we chose the four‐cluster partitioning since its inertia quotient corresponded to an elbow point in the graph and due to the number of care unit in each cluster. Cramer's contingency coefficients (presented in Table 2) were then computed to indicate the strength of association between each indicator and all clusters (Liebetrau, 1983). The clusters grouped the care units according to similarities in their staffing indicators, thus forming distinct configurations of nurse staffing, which were used to generate nurse staffing groups. The statistical analysis was performed using SPSS version 23 and SPAD version 8.

**Table 2:** Characteristics of the four identified models according to the three staffing dimensions

Indicators	Total, *N* (%) (*N* = 40)	Least resourced model, % (*N* = 6)	Moderately resourced basic model, % (*N* = 13)	Moderately resourced professional model, % (*N* = 10)	Most resourced model, % (*N* = 11)	Cramer's contingency coefficients
Resource levels						
Patient‐to‐nurse ratio						
Lower	17 (42.5)	0.00	15.38	60.00	81.82	0.752*
Average	18 (45)	16.67	84.62	40.00	18.18	
Higher	5 (12.5)	83.33	0.00	0.00	0.00	
Ratio of patients to auxiliary nurses and nurse aides						
Lower	4 (10)	0.00	7.69	0.00	27.25	0.602*
Average	22 (55)	100.00	92.31	20.00	18.20	
Higher	14 (35)	0.00	0.00	80.00	54.55	
Overtime of RNs with bachelor's degree						
Lower	28 (70)	66.67	84.62	50.00	72.73	0.287
Higher	12 (30)	33.33	15.38	50.00	27.27	
Overtime of RNs without bachelor's degree						
Lower	29 (72.5)	66.67	92.31	40.00	81.82	0.459*
Higher	11 (27.5)	33.33	7.69	60.00	18.18	
Overtime of assistant head nurses						
Lower	35 (87.5)	83.33	100.00	60.00	100.00	0.511*
Higher	5 (12.5)	16.67	0.00	40.00	0.00	
Staff mix						
Proportion of RNs with bachelor's degree						
Lower	7 (17.5)	33.33	0.00	40.00	9.09	0.482*
Average	25 (62.5)	66.67	84.62	60.00	36.36	
Higher	8 (20)	0.00	15.38	0.00	54.55	
Proportion of RNs without bachelor's degree						
Lower	9 (22.5)	100.00	0.00	10.00	18.18	0.612*
Average	21 (52.5)	0.00	84.62	60.00	36.36	
Higher	10 (25)	0.00	15.38	30.00	45.46	
Proportion of assistant head nurses						
Lower	5 (12.5)	0.00	0.00	0.00	45.46	0.518*
Average	22 (55)	33.33	84.62	50.00	36.36	
Higher	13 (32.5)	66.67	15.38	50.00	18.18	
Proportion of auxiliary nurses						
Lower	19 (47.5)	0.00	7.69	70.00	100.00	0.601*
Average	6 (15)	16.67	30.77	10.00	0.00	
Higher	15 (37.5)	83.33	61.54	20.00	0.00	
Proportion of nurse aides						
Lower	8 (20)	0.00	0.00	30.00	45.46	0.366
Average	25 (62.5)	66.67	76.92	60.00	45.46	
Higher	7 (17.5)	33.33	23.08	10.00	9.08	
Support staff						
No	5 (12.5)	0.00	0.00	0.00	45.46	0.614*
Yes	35 (87.5)	100.00	100.00	100.00	54.55	
Team stability						
Absenteeism of RN with bachelor's degree						
Lower	21 (52.5)	66.67	69.23	30.00	45.50	0.264
Average	13 (32.5)	16.67	15.39	50.00	45.50	
Higher	6 (15)	16.60	15.39	20.00	9.00	
Absenteeism of RNs without bachelor's degree						
Lower	9 (22.5)	50.00	15.36	0.00	36.40	0.551*
Average	12 (30)	50.00	23.10	0.00	54.50	
Higher	19 (47.5)	0.00	61.54	100.00	9.10	
Absenteeism of assistant head nurses						
Lower	20 (50)	66.67	23.08	30.00	90.90	0.444*
Average	12 (30)	33.33	38.46	50.00	0.00	
Higher	8 (20)	0.00	38.46	20.00	9.10	
Supplemental agency nurses						
No	35 (87.5)	33.33	100.00	100.00	90.91	0.698*
Yes	5 (12.5)	66.67	0.00	0.00	9.09	

Abbreviation: RN, Registered Nurse.

* The associated *p* value is lower than 0.05.

## 5 RESULTS

Four distinct configurations arose from the data analysis. As previously mentioned, Cramer's contingency coefficients were computed; for most indicators, the coefficient was over 0.5, indicating a strong association between the indicator and the classifications (Table 2). The four nurse staffing groups that were defined based on these configurations exhibit varying levels of coverage in providing adequate nurse staffing to respond to healthcare needs.

### 5.1 The least resourced group

This group represented six care units and included all short‐term geriatric care units in the sample as well as some medical and surgical care units. Resource level indicators showed a high patient‐to‐nurse ratio and an average ratio of patients to auxiliary nurses and nurse aides. The overtime hour rates were low for all types of nursing staff. The staff mix presented a high proportion of auxiliary nurses and an average proportion of nurse aides. Also, there was a high proportion of assistant head nurses, a low proportion of RNs without bachelor's degree and an average proportion of RNs with bachelor's degrees. All units were provided with support staff. In terms of team stability, absenteeism rates were low for RNs with bachelor's degrees and assistant head nurse and low to average for RNs without bachelor's degree. Two‐thirds of the care units in this group used agency staff at least once during the target data collection period.

### 5.2 The moderately resourced basic group

This group was composed of 13 care units, mainly medical or surgical facilities. In terms of resource levels, both the patient‐to‐nurse ratio and the ratio of patients to auxiliary nurses and nurse aides were average. Overtime rates for all types of nursing staff were low. This was consistent with the staff mix indicators, which showed that each type of personnel was represented in average proportion, except for a higher proportion of auxiliary nurses. All units had access to support staff. Team stability indicators revealed high absenteeism rates among RNs without bachelor's degree and average to high absenteeism rates for assistant head nurses. This group presented the highest overall absenteeism rate in the studied sample. No care unit in this group used supplemental agency staff, which offset the negative effect of high absenteeism rates on team stability, resulting in only moderate instability for this group.

### 5.3 The moderately resourced professional group

This group contained 10 care units, including all the sample's psychiatry wards, almost all of its maternity wards and a few critical care units. Resource level indicators showed a low patient‐to‐nurse ratio and a high ratio of patients to auxiliary nurses and nurse aides. This group had generally high overtime needs. Indeed, overtime rates for all types of nurses were among the highest in the entire sample. about the staff mix dimension, every care unit had access to support staff. Proportions of each type of personnel on the team were average, except for auxiliary nurses, who were present in low proportion and assistant head nurses, who were present in average to high proportions on the nursing team. In terms of team stability, none of the units had required supplemental agency nurses during the period studied. There were moderate to high rates of absenteeism, indicating significant instability in these care units.

### 5.4 The most resourced group

This group was composed of 11 units that offered tertiary care; they were either critical care units or specialized medical care units. In terms of resource levels, the patient‐to‐nurse ratio was categorized as low and the ratio of patients to auxiliary nurses and nurse aides as high. Overtime rates were low for all types of nurses. Staff mix was consistent with the low patient‐to‐nurse ratio, showing high proportions of RNs (with and without bachelor's degree) on the nursing teams. There were low proportions of auxiliary nurses and assistant head nurses and low to average proportions of nurse aides in the nursing teams. In this group, 45% (*N* = 5) of the care units showed an absence of support staff. Concerning team stability, only one care unit had used supplemental agency nurses once or more. Team instability in this group was average, reflecting the low absenteeism rates for assistant head nurse and low to average absenteeism rates among RNs with and without bachelor's degree.

## 6 DISCUSSION

The goal of the study was to identify the staffing groups in acute care facilities of a region and their associated staffing characteristics. Four groups emerged from the data analysis: the least resourced group, a moderately resourced basic group, a moderately resourced professional group and the most resourced group. While this study has been merely descriptive, other related works can provide insights on potential effects that can be associated with the different staffing groups.

### 6.1 A group with highly at‐risk quality of care

The least resourced group fit into the lowest categories of all three dimensions studied. This translated into three major characteristics: low levels of all types of personnel, significant use of less qualified staff and instability as evidenced by significant use of agency staff.

Previous studies have demonstrated that these three characteristics are associated with poor quality of care. Low staffing levels and intensive use of less qualified personnel are associated with higher risks of adverse events for patients (Aiken et al., 2011, 2014; Duffield et al., 2011; Estabrooks, Midodzi, Cummings, Ricker, & Giovannetti, 2005; Hart & Davis, 2011). Some studies have established a link between care team instability and lower quality of care (Estabrooks et al., 2005; Hart & Davis, 2011; Institute of Medecine [IOM], 2003).

The staffing characteristics of the least resourced group corresponded to what was defined as a “basic functional group” in another study and considered as the farthest from the ideal staffing group (Dubois et al., 2012). The care units in this group were unstable and responded poorly to staffing needs in the face of healthcare demand.

This group's characteristics were likely to lead to a negative chain reaction in care units. A lack of resources potentially promotes and is perpetuated by, a negative work environment, which can lead to more instability and to difficulties in attracting and retaining staff, which could lead to lower quality of care (Aiken, Shang, Xue, & Sloane, 2013; Hart & Davis, 2011; IOM, 2003; Keogh, 2013).

Care units in the least resourced group were short‐term geriatric hospitalization units and general medical‐surgical units. This group suggests that in a context of resource constraints, minimal priority was accorded to this type of general care unit. They had to cope with low resource levels and significantly less qualified personnel, despite the potential risks this posed to quality of care. Dubois et al. (2012) state that this type of group seems to represent an alternative for some health organizations to respond to economic and market constraints.

### 6.2 A group with poor quality guarantees

The care units of the moderately resourced basic group were assessed as moderate for most of the studied variables (according to the categorizations defined in Table 1). The three main characteristics of this group were moderate resource levels, moderate staff mix (as shown by moderate proportions of all types of personnel except auxiliary nurses) and instability, as demonstrated by the highest absenteeism rate in the entire sample. These characteristics were substantially similar to those of the “adaptive functional” group identified in another study (Dubois et al., 2012) where the facilities also relied heavily on the use of less qualified staff. These facilities also seemed to cope to the moderate resources levels available in relation with the “economic and labour‐market constraints” (Dubois et al., 2012).

Studies have associated this group's characteristics with higher risks and poor patient outcomes. As mentioned previously, research has found that high proportions of less qualified personnel in the care team increase the occurrence of adverse effects and mortality (Aiken et al., 2011; Aiken, Clarke, Sloane, Sochalski, & Silber, 2002; Dubois & Singh, 2009; Estabrooks et al., 2005; Hart & Davis, 2011). Absenteeism is also associated with factors that lower quality of care, such as increased workload for the remaining nurses (Bogossian, Winters‐Chang, & Tuckett, 2014; Tourangeau, Wong, Saari, & Patterson, 2015) and overtime (Bae, 2013; Lobo et al., 2013).

The care units of the moderately resourced basic group were essentially general medical‐surgical units. This observation confirms the lack of guidelines about staffing and resulting staffing variations for the same types of unit in a same organization. In this case, general medical care units are distributed in two different groups: the least resourced group and the moderately resourced basic group. In this second group, the care units had to cope with moderate resource levels and low proportions of qualified personnel on care teams. These units faced high absenteeism rates among nurses. It can be hypothesized that these staffing conditions were linked to recent reorganization and rationalization initiatives that many healthcare systems experienced, and which often target these types of units (Aiken et al., 2011, 2002).

### 6.3 A group approaching general staffing recommendations

The moderately resourced professional group was characterized by low patient‐to‐nurse ratios, high overtime rates, more qualified than less qualified personnel and team instability (manifesting in moderate to high absenteeism).

As previously mentioned, higher proportions of qualified personnel and lower proportions of less qualified personnel among a care team are associated with lower risk of adverse events and significantly better patient outcomes, resulting in enhanced quality of care (Aiken et al., 2011, 2002, 2014; Dubois & Singh, 2009; Estabrooks et al., 2005; Hart & Davis, 2011). However, these positive effects are potentially offset by other, unfavourable characteristics: intensive use of overtime (Bae, 2012; Bae, Brewer, & Kovner, 2012; Drebit, Ngan, Hay, & Alamgir, 2010) and high absenteeism rates. Given the low patient‐to‐nurse ratios, the moderate proportions of personnel and the high use of overtime, we could hypothesize that these care units were able to maintain sufficient resource levels at least partly by making use of overtime.

The moderately resourced professional group was composed of specialized medical units, psychiatric wards and critical care units. They had higher levels of personnel than the two previous groups. This could be linked to the recommendations for these types of care units, which call for high proportions of qualified personnel (MSSS, 2013).

### 6.4 The staffing group closest to the theoretical ideal

The most resourced group represented the best possible categories for each studied variable. The first major characteristic of this group was high resource levels, as evidenced by the lowest patient‐to‐nurse ratio in the sample. Second, it presented low proportions of less qualified personnel and high proportions of qualified personnel‐care units in this group had the highest proportions of RNs with bachelor's degrees on their nursing teams. Third, there was low instability, as demonstrated by the absenteeism rates, which were among the lowest in the entire sample for all types of nurses, except RNs with a bachelor's degree.

This group was the closest to the theoretical ideal nurse staffing group. Indeed, the three characteristics of this group have been associated with lower risks of adverse events for patients (Aiken et al., 2011, 2014; Dubois & Singh, 2009; Estabrooks et al., 2005; Hart & Davis, 2011).

The most resourced group contained most of the critical care units in the studied sample as well as some medical units offering tertiary care. This group seemed to follow the recommendation issued by the MSSS, according to which these types of care units should have high proportions of qualified personnel and low proportions of less qualified personnel (MSSS, 2013). Based on its staffing characteristics, this group sought to maximize the quantity and quality of care delivered, while minimizing risks.

In the light of the four identified staffing groups, the results and the descriptive information presented in this study can provide important guidance for nurse workload planning. However, we recommend to hospital centres’ decision‐makers to further analyse the staffing data they systematically collect. This could ensure appropriate staffing interventions based on concrete data concerning their own staffing situation. Moreover, it would be relevant for future research to assess nurse staffing in its entirety to avoid a fragmented vision of the concept.

### 6.5 Limitations

The broad range of variables involved in nurse staffing explains the presence of variation between and within staffing groups. Internal variations might be partially explained by the fact that the four staffing groups were derived from real conditions observed for each care unit. Typical ideal staffing groups based on care unit's operational needs and settings could differ from the configurations obtained empirically in this study. This could also reflect another limitation of this study, namely that due to a recent reform of the Quebec healthcare system that entailed significant merging and reorganization of health establishments across the province, some care units might have been undergoing a change process affecting their staffing variables or transitioning towards another staffing group. As these changes could have been occurring at different rates for all staffing dimensions during the target period, even in a same care unit, they could have resulted in inconsistencies in the studied variables.

Another limitation of the study was the unavailability of some data, which meant that the evaluation of “resource levels” and “staff mix” dimensions was not as thorough as it could have otherwise been. The unavailability of data was also a limitation concerning the patient‐to‐staff ratio. This ratio was developed in two indicators since the auxiliary nurses and nurse’ aides worked hours were not computed.

## 7 CONCLUSION

To our knowledge, this study was the first empirical description of staffing groups in Quebec. The naturally present variations in care unit staffing characteristics lent themselves to a classification resulting in four staffing groups, some of which were more potent in meeting staffing needs than others. This study showed that health organizations used a variety staffing groups, most of which contained ingredients for low quality and security of care.

The results of this study provided new data in terms of empirical groups of nurse staffing in acute care in Quebec. Similarly to other industrialized countries, care teams in Quebec are composed of different groups of personnel: Registered Nurses, licensed practical nurses and assistive staff. Thus, the results could be useful for healthcare managers in similar contexts. This study's findings were innovative and could be used to plan and manage nursing resource and staffing interventions. They also provided a current portrait of nurse staffing in acute care in Quebec, which can contribute significantly to the first step necessary for any optimization efforts in the health network, namely the descriptive phase.

A great strength of this study was the population studied. Except for three care units that did not meet the inclusion criteria, the sample included all acute care units in the region under study. Thus, we can presume that the results could be transferable to similar health facilities in similar contexts. Further research could investigate other care settings, considering all nurse staffing variables concurrently while evaluating their relationship with patient outcomes.

## CONFLICT OF INTEREST

No conflict of interest has been declared by the author(s).

## AUTHORS’ CONTRIBUTIONS

MT, CAD, RBDS and AP: Conception and design, or acquisition of data, or analysis and interpretation of data and agreed to be accountable for all aspects of the work in ensuring that questions related to the accuracy or integrity of any part of the work are appropriately investigated and resolved. MT, CAD and RBDS: Drafting the manuscript or revising it critically for important intellectual content and final approval of the version to be published. Each author should have participated sufficiently in the work to take public responsibility for appropriate portions of the content.
